# Study on restless leg syndrome and its relationship with quality of sleep among the general population of Mangalore, India

**DOI:** 10.1186/s41983-022-00544-z

**Published:** 2022-09-15

**Authors:** Nitin Joseph, Sooraj Suresh, Satwiki Prasad, Swaraj Mandar Malwee, Anand Brittas, Vedant Gupta

**Affiliations:** 1 Department of Community Medicine, Kasturba Medical College, Mangalore, Manipal Academy of Higher Education, Manipal, India; 2 Kasturba Medical College, Mangalore, Manipal Academy of Higher Education, Manipal, India

**Keywords:** Restless leg syndrome, Severity, Determinants, Quality of sleep, Population-based study

## Abstract

**Background:**

Restless leg syndrome (RLS) is a common neurological morbidity. It is, however, a frequently underdiagnosed medical condition. This study was hence done to assess the occurrence and severity of RLS among participants and to study its determinants and its association with quality of sleep. This was a cross-sectional study conducted among the general population of Mangalore in July 2021. Data were collected using a Google Form. The International Restless Legs Syndrome Study Group Rating Scale was used to diagnose RLS and its severity. The Pittsburgh Sleep Quality Index (PSQI) was used to assess sleep quality.

**Results:**

The prevalence of RLS among the 202 participants was 24(11.9%). Among them, 5 were already diagnosed with RLS. Their mean age at onset was 40.4 ± 25.3 years. Among the rest 197 participants, 19(9.6%) were newly diagnosed with RLS. The severity of RLS was mild, moderate and severe among 7(36.8%), 9(47.4%) and 3(15.8%) participants, respectively. Five (26.3%) of the 19 newly diagnosed participants were identified as RLS sufferers. In multivariable analysis, the presence of diabetes mellitus and family history of RLS were  associated with the presence of RLS among the participants. The mean Global PSQI value was 5.0 ± 3.1. Sleep latency was prolonged (*p* = 0.001), and sleep disturbances (*p* = 0.01) were higher among participants newly diagnosed with RLS (*n* = 19) compared to those without RLS (*n* = 178). Subjective sleep quality was poor (*p* = 0.038), and sleep disturbances (*p* = 0.016) were more among participants with severe degree RLS.

**Conclusions:**

The prevalence of RLS in the present study was higher than that reported in previous Indian studies. Unpleasant sensations in RLS affected sleep initiation and maintenance among the affected. A multi-disciplinary approach is required to control its determinants and address other sleep-related problems among the RLS affected population.

## Background

Restless leg syndrome (RLS) is a common neurological morbidity. It affects as many as 4–29% of the general population in developed countries [1]. It is characterized by unpleasant sensations in the limbs relieved by movement. It is associated with sleep disturbances, anxiety, depression and deterioration in quality of life [2]. It thus has a significant social burden.

Despite these related problems, it is still a frequently underdiagnosed medical condition in India [3, 4]. Delay in diagnosis occurs probably due to minimal sensitization of this disease among medical practitioners. This may be why it is often wrongly diagnosed as an anxiety disorder or as a manifestation due to underlying exertion or fatigue [5]. There have also been reports of non-seeking of medical help by the patients due to ignorance of RLS [5]. Hence, there is a need to increase awareness about RLS among medical professionals and the general population, so that people suffering from it can be diagnosed early and managed. The understanding of its determinants is also very minimal, and therefore, a better understanding of its determinants will help to frame suitable preventive measures.

Prior studies on RLS were not comprehensively conducted. Some studied the occurrence of RLS only among people aged 40 years and above, while others studied its occurrence in patients with only specific diseases, such as diabetes mellitus and depression [6–8].

Hence, it was necessary to conduct this study to address these limitations. It would also help to improve the understanding of people toward RLS, its determinants, and its adverse health consequences. With this background, the present study was conducted to assess the occurrence and severity of RLS among participants and to study its determinants and its association with quality of sleep.

## Methods

This cross-sectional study was done in July 2021. The institutional ethics committee gave the approval to conduct this study. The approval number was IEC KMC MLR 05-2020/200(C).

The formula *Z*_*α*_^2^*PQ*/*d*^2^ was used to calculate the sample size. In a study done in Pakistan [5], 23.6% of participants had RLS using the International Restless Legs Syndrome Study Group Rating Scale (IRLSSG) screening tool. The minimal sample size at 95% confidence intervals and 6% absolute precision was calculated as 193.

The questionnaire used for data collection was designed as a Google Form. It was content validated and pilot tested before its use in the present study. Considering the threat due to the ongoing COVID-19 pandemic, data were collected by sharing the questionnaire link using WhatsApp and email among the general population of Mangalore. The participants were hence recruited in this study using non-random sampling method. The first page of the questionnaire contained the study information sheet and the consent form.

Participants aged 18 years and above were included in this study. Those who did not give consent for participation, and those aged below 18 years of age were excluded from this study.

The first part of the questionnaire enquired sociodemographic details, total monthly family income, number of family members, known status of RLS, history of other comorbidities, history of using substances of abuse, self-reported height and weight and recent blood investigation reports of fasting blood sugar (FBS), glycosylated haemoglobin, serum creatinine, and blood urea nitrogen values among the participants. The second part constituted the IRLSSG screening tool. Initial four questions of this tool were meant to diagnose RLS based on the symptoms experienced in the preceding 1 week. The subsequent 10 questions were designed in a five-point Likert scale with scores ranging from 0 to 4. These were meant to assess the severity among those newly diagnosed with RLS over the preceding week. The severity was considered “none, mild, moderate, severe and very severe” if the cumulative score was 0, 1–10, 11–20, 21–30 and 31–40, respectively. The third part of the questionnaire constituted the Pittsburgh Sleep Quality Index (PSQI). This assessed the quality of sleep over the past 1 month. It had 10 questions designed in a four-point Likert scale scored from 0 to 3. These questions assessed seven components under sleep quality. The total of the seven component scores gave the Global PSQI score, which ranged from 0 to 21 (no difficulty to severe difficulty in sleeping).

The internal consistency Cronbach’s alpha of the IRLSSG screening tool used in this study was found to be 0.95, and of the PSQI was found to be 0.7.

The early onset RLS was defined as patients diagnosed with RLS before 45 years [9].

RLS sufferers were those participants who reported both frequencies of symptoms at least twice a week and experienced distress due to symptoms of RLS of the moderate or severe degree over the past 1 week [10].

In this study, current users of substances of abuse were defined as those who used tobacco (either in smoked or smokeless form) or alcohol within the recent 1 month [11].

Body mass index (BMI) was categorized based on the Asia–Pacific classification. Socioeconomic status (SES) was classified based on the Modified BG Prasad classification of 2021.

Data were analyzed using SPSS Statistics for Windows, Version 25.0; IBM Corp, Armonk, NY, USA. Statistical tests such as the Independent samples *t* test, Chi-Square test, Fisher’s exact test, and binary logistic regression analysis were applied. *p* ≤ 0.05 was set as cut off to state statistical significant association.

## Results

The mean age of the participants was 29 ± 13 years. It ranged from 18 to 84 years (Table 1).

**Table 1:** Sociodemographic distribution of study participants

Characteristics	Number	Percentage
Age interval (years)		
≤ 20	30	14.8
21–30	123	60.9
31–40	9	4.5
41–50	13	6.4
51–60	23	11.4
> 60	4	2.0
Gender		
Males	93	46.0
Females	109	54.0
Occupation		
Professional	35	17.3
Semi-professional	28	13.9
Student	116	57.4
Skilled workers	12	5.9
Semi-skilled workers	3	1.5
Unskilled worker	5	2.5
Housewives	3	1.5
Education (*n* = 177)		
Post-graduate	45	25.4
Graduate	125	70.6
Pre-university course	4	2.3
High school	3	1.7
Marital status		
Unmarried	145	71.8
Married	55	27.2
Divorced	1	0.5
Widowed	1	0.5
Socioeconomic status (*n* = 10)		
Class 1	8	80.0
Class 2	2	20.0
Place of residence		
Urban	169	83.7
Semi-urban	33	16.3
Total	202	100.0

Among the participants, 2(1%) had a parental history of consanguineous marriage. Thirty-seven (18.3%) had one or other comorbidities. This comprised of type II diabetes mellitus among 17(8.4%), hypertension among 15(7.4%), asthma among 7(3.5%), anaemia among 6(3%), snoring, dyslipidaemia and hypothyroidism among 4(2%) participants each, diabetic foot among 3(1.5%), rheumatoid arthritis and hyperthyroidism among 2(1%) participants each and type I diabetes mellitus, polycystic ovarian disease, osteoarthritis, and obsessive–compulsive disorders among 1 participant each out of the 202 participants. Of the total participants, 21(10.4%) were current smokers, 15(7.4%) were ex-smokers, 5(2.5%) each were current tobacco chewers and ex-tobacco chewers, 58(28.7%) were current alcohol users, and 26(12.9%) were ex-alcohol users.

Among the 18 patients with either Type I or II diabetes mellitus, recent FBS values were on the higher side among 8, recent HbA1c values were on the higher side among 5, blood urea nitrogen values were on the higher side among 2, and serum creatinine values were on the higher side in 1 patient. The median duration of diabetes mellitus was 8.5 years (IQR 2.5, 19.75 years).

Out of the 18 diabetic patients, 11(61.1%) felt that their blood sugars were well in control, 5(27.7%) felt that it was somewhat in control, 1(5.6%) felt that it was in little control, and 1(5.6%) felt that it was not at all in control.

The prevalence of RLS out of the total 202 participants was 24(11.9%). Among them, 5 were known patients with RLS. Their mean age at onset was 40.4 ± 25.3 years. Four of them had early age at the onset of RLS.

Among the known patients with RLS, both the patients with a family history of RLS had early age at the onset of RLS. In comparison, out of 3 patients without a family history of RLS among the known patients with RLS, 2(66.7%) had early age at the onset of RLS (*p* = 1).

Out of 13(6.4%) participants with a family history of RLS out of the total participants, 3 reported it to be present in their father, 6 in their mother, 1 in both father and brother, 1 in grandfather, 1 in sister, and 1 in uncle (Table 2).

**Table 2:** Restless leg syndrome and its characteristics among the study participants

Characteristics	Number	Percentage
Known case of RLS (*n* = 202)	5	2.5
Exacerbating factors of RLS (*n* = 5)*		
Exercise	4	80.0
Drugs	3	60.0
Treatment taken for RLS (*n* = 5)		
Ayurveda	3	60.0
Homeopathy	2	40.0
Family history of RLS (*n* = 202)		
Present	13	6.4
Positive for RLS using the screening tool (*n* = 197)	19	9.6
Distribution of parameters indicating the severity of RLS in the past 1 week among the newly diagnosed RLS participants (*n* = 19)		
RLS discomfort in arms or legs		
Mild	12	63.2
Moderate	5	26.3
Severe	2	10.5
RLS symptoms prompt the need to move around to get relief (*n* = 19)		
Mild	11	57.9
Moderate	5	26.3
Severe	3	15.8
Sleep disturbances due to RLS symptoms (*n* = 19)		
None	7	36.9
Mild	5	26.3
Moderate	5	26.3
Severe	2	10.5
Tiredness or sleepiness due to RLS symptoms (*n* = 19)		
None	7	36.9
Mild	5	26.3
Moderate	5	26.3
Severe	2	10.5
Self-perceived severity of RLS as a whole (*n* = 19)		
Mild	13	68.4
Moderate	6	31.6
Impact of RLS symptoms on day-to-day affairs (*n* = 19)		
None	10	52.6
Mild	5	26.3
Moderate	4	21.1
Influence of RLS symptoms on mood disturbance (*n* = 19)		
None	9	47.4
Mild	5	26.3
Moderate	3	15.8
Severe	2	10.5
Relief of RLS (arms or legs) by moving around (*n* = 19)		
Complete relief/almost complete relief	10	52.6
Moderate relief	3	15.8
Slight relief	5	26.3
No relief	1	5.3
Frequency of RLS symptoms (*n* = 19)		
1 day a week or less	12	63.2
2–3 days a week	2	10.5
4–5 days a week	3	15.8
6–7 days a week	2	10.5
Duration of RLS symptoms on an average per day (*n* = 19)		
< 1 h per day	14	73.7
1 to 3 h per day	3	15.8
3 to 8 h per day	2	10.5

*RLS* Restless leg syndrome

^*^Multiple responses

Out of the 24 participants, both previously diagnosed and newly diagnosed with RLS, 7(29.2%) had a family history of RLS.

Out of the 197 participants who were not diagnosed with RLS before, 19(9.6%) were found positive for RLS using the screening tool (Table 2).

The severity of RLS among the 19 participants newly diagnosed with RLS was of mild, moderate, and severe degree among 7(36.8%), 9(47.4%), and 3(15.8%), respectively, based on the ten parameters indicating the severity of RLS listed in Table 2.

Of the newly diagnosed participants, 6(31.6%) reported moderate degree of symptoms, and 7(36.8%) reported frequency of symptoms at least twice a week (Table 2).

Five (26.3%) of the 19 newly diagnosed participants fulfilled both these criteria and were identified as RLS sufferers. The prevalence of RLS sufferers was 5(2.5%) out of the 197 participants.

The presence of RLS (both newly/previously diagnosed) was seen significantly more among participants aged > 30 years, those married, those with comorbidities, such as diabetes mellitus and hypertension, and those with a family history of RLS in the univariate analysis (Table 3).

**Table 3:** Association between various determinants and restless leg syndrome status among the participants (*n* = 202)

Characteristics	RLS positive on screening/known case of RLS No. (%)	RLS negative No. (%)	Total	UOR (95% CI)	AOR (95% CI)	*p* value
Age group (years)						
> 30	10(20.4)	39(79.6)	49			
≤ 30	14(9.2)	139(90.8)	153			
			*X*^2^ = 4.493, *p* = 0.034	2.546(1.05–6.173)	0.636(0.087–4.636)	0.656
Marital status						
Married	11(20.0)	44(80.0)	55			
Unmarried/Divorced/Widowed	13(8.8)	134(91.2)	147			
			*X*^2^ = 4.758, *p* = 0.0292	2.577(1.077–6.164)	1.832(0.28–11.976)	0.527
Presence of diabetes mellitus						
Yes	8(44.4)	10(55.6)	18			
No	16(8.7)	168(91.3)	184			
			*X*^2^ = 20.0, *p* < 0.001	8.4(2.905–24.288)	6.696(2.123–21.118)	< 0.001
Presence of hypertension						
Yes	5(33.3)	10(66.7)	15			
No	19(10.2)	168(89.8)	187			
			*X*^2^ = 7.122, *p* = 0.008	4.421(1.367–14.297)	1.204(0.243–5.974)	0.82
Family history of RLS						
Present	7(53.8)	6(46.2)	13			
Absent	17(9)	172(91)	189			
			*X*^2^ = 23.371, *p* < 0.001	11.804(3.559–39.147)	9.254(2.555–33.512)	< 0.001
Total	24	178	202			

*RLS* Restless leg syndrome, *UOR* unadjusted odds ratio, *AOR* adjusted odds ratio, *CI* confidence intervals

There was no association of RLS status with gender (*p* = 0.647), occupation (*p* = 0.987), educational status (*p* = 0.226), number of family members (*p* = 0.592), BMI (*p* = 0.424), SES (*p* = 1), residential status (*p* = 0.07), presence of comorbidities (*p* = 0.409), chewing tobacco status (*p* = 0.3377), smoking cigarettes status (*p* = 1), drinking alcohol status (*p* = 0.228) among the participants and the self-perceived control status of blood sugar among the diabetic patients (*p* = 0.1534).

In multivariable analysis, the presence of diabetes mellitus (*p* < 0.001) and family history of RLS (*p* < 0.001) were associated with the presence of RLS among the participants (Table 3).

Out of 12 female patients newly diagnosed with RLS, 3(25%) had severe degree RLS. Out of 7 male patients newly diagnosed with RLS, none had severe degree RLS (*p* = 0.263).

There was no association of sociodemographic variables, history of usage of substances of abuse, presence of comorbidities, duration of diabetes mellitus, and abnormalities in the recent blood investigation report with the severity of RLS among the participants.

Among the five newly diagnosed RLS participants with a family history of RLS, 2(40%) were RLS sufferers. Out of the rest 14 newly diagnosed RLS participants without a family history of RLS, 3(21.4%) were RLS sufferers (*p* = 0.57).

The Global PSQI score ranged from 0 to 17. Its mean value was 5.0 ± 3.1.

Sleep latency was prolonged (*p* = 0.001) and sleep disturbances (*p* = 0.01) were higher among participants newly diagnosed with RLS (*n* = 19) compared to those without RLS (*n* = 178) (Table 4).

**Table 4:** Association between the presence of restless leg syndrome with quality of sleep among the participants (*n* = 197)

Sleep quality parameters	RLS screening status	Number of participants	Mean score ± SD over the past 1 month	95% CI of mean score
Subjective sleep quality	Positive	19	0.842 ± 0.765	− 0.371 to 0.246
	Negative	178	0.904 ± 0.635	
				*t* = 0.399, *p* = 0.69
Sleep latency	Positive	19	1.74 ± 0.81	0.3 to 1.22
	Negative	178	0.98 ± 0.8	
				*t* = 3.26, *p* = 0.001
Sleep duration	Positive	19	0.737 ± 0.653	− 0.374 to 0.297
	Negative	178	0.775 ± 0.61	
				*t* = − 0.226, *p* = 0.821
Habitual sleep efficiency	Positive	19	0.838 ± 0.579	− 0.308 to 0.466
	Negative	178	0.8 ± 0.5	
				*t* = 0.402, *p* = 0.688
Sleep disturbances	Positive	19	1.16 ± 0.69	0.0892 to 0.642
	Negative	178	0.79 ± 0.57	
				*t* = 2.608, *p* = 0.01
Usage of sleep medications	Positive	19	0.46 ± 0.105	− 0.309 to 0.228
	Negative	178	0.57 ± 0.146	
				*t* = 0.3, *p* = 0.765
Daytime dysfunction	Positive	19	0.842 ± 0.765	− 0.285 to 0.453
	Negative	178	0.776 ± 0.758	
				*t* = 0.447, *p* = 0.655
Global PSQI score	Positive	19	6.0 ± 2.87	− 0.281 to 2.573
	Negative	178	4.854 ± 3.01	
				*t* = 1.584, *p* = 0.115

*RLS* Restless leg syndrome, *SD* standard deviation, *CI* confidence intervals, *PSQI* Pittsburgh Sleep Quality Index

The Global PSQI among the participants with mild degree RLS (*n* = 7) was 5.4 ± 2.3 and among those with moderate or severe degree RLS (*n* = 12) was 6.3 ± 3.2 (*t* = 0.653, *p* = 0.523).

Subjective sleep quality was poor (*p* = 0.038), and sleep disturbances (*p* = 0.016) were more among participants with severe degree RLS (Table 5).

**Table 5:** Association between restless leg syndrome (RLS) severity status and quality of sleep among the newly diagnosed participants with RLS (*n* = 19)

Sleep quality parameters	RLS severity status	Number of participants	Mean score ± SD over the past 1 month	95% CI of mean score
Subjective sleep quality	Severe degree	3	1.7 ± 0.6	0.063 to 1.896
	Not severe	16	0.7 ± 0.5	
				*t* = 2.254, *p* = 0.038
Sleep latency	Severe degree	3	2.0 ± 1.0	− 0.776 to 1.401
	Not severe	16	1.7 ± 0.8	
				*t* = 0.606, *p* = 0.553
Sleep duration	Severe degree	3	1.0 ± 0.7	− 0.565 to 1.19
	Not severe	16	0.7 ± 0.5	
				*t* = 0.751, *p* = 0.463
Habitual sleep efficiency	Severe degree	3	0 ± 0	− 1.776 to 0.401
	Not severe	16	0.9 ± 0.7	
				*t* = 1.332, *p* = 0.2
Sleep disturbances	Severe degree	3	2.0 ± 1.0	0.211 to 1.789
	Not severe	16	1.0 ± 0.5	
				*t* = 2.675, *p* = 0.016
Usage of sleep medications	Severe degree	3	0 ± 0	− 0.748 to 0.498
	Not severe	16	0.1 ± 0.1	
				*t* = 0.423, *p* = 0.678
Daytime dysfunction	Severe degree	3	1.0 ± 0.8	− 0.853 to 1.228
	Not severe	16	0.8 ± 0.6	
				*t* = 0.38, *p* = 0.708
Global PSQI score	Severe degree	3	7.7 ± 4.0	− 1.804 to 5.762
	Not severe	16	5.7 ± 2.6	
				*t* = 1.104, *p* = 0.285

*RLS* Restless leg syndrome, *SD* standard deviation, *CI* confidence intervals, *PSQI* Pittsburgh Sleep Quality Index

The mean subjective sleep quality scores among RLS sufferers (*n* = 5) was 1.4 ± 0.55 in comparison with 0.745 ± 0.643 among non-sufferers (*n* = 14) [95% CI − 0.0162 to 1.53, *p* = 0.054].

## Discussion

The prevalence of RLS in the present study was found to be 11.9% compared to 5.5% [12], 8.4% [13], 13.7% [6] and 23.6% [5] reported in studies done in other parts of the world. However, in other Indian studies, its prevalence was 2.1% [3] and 2.9% [4]. Therefore, its prevalence was much higher in the present study compared to previous studies done in India. This could be because of varying age distribution, family history of RLS and morbidity pattern among the participants of previous Indian studies compared to participants in this study. In the study done by Panda and colleagues, the age distribution of participants ranged from 16 to 55 years compared to 18 to 84 years in the present study [4]. In the other study done by Rangarajan and colleagues, the proportion of patients with diabetes mellitus and hypertension was 4.9% and 4.7%, respectively, in comparison with 8.9% and 7.4% observed in the present study [3]. In addition, family history of RLS was reported by 0% [4] and 18.5% [3] participants in these studies compared to 29.2% reported by participants in the present study.

Determinants independently associated with RLS status among the participants in the present study were family history of RLS and the presence of diabetes mellitus. This highlights the role of genetic factors and lifestyle diseases in the occurrence of RLS. Analytical studies in genetics have identified 20 single nucleotide polymorphisms in 19 loci on the chromosomes related to RLS [14].

However, there was no association between family history of RLS and early age at the onset of RLS among the previously diagnosed RLS patients in this study.

In previous studies, young age [6], increasing age [5, 12, 15, 16], male gender [13, 16], female gender [5, 15], low educational status [3, 5, 15], poor SES [3], history of consanguinity [13], history of smoking [5, 13], history of alcohol usage [3], higher BMI [15], presence of depression [6, 12], anxiety [6], stress [5, 6], gastritis [15], anaemia [15], hypertension [15], diabetes mellitus [13], bronchial asthma [13] and habitual snoring practice [13] were associated with the presence of RLS among the participants.

The proportion of RLS sufferers among the newly diagnosed RLS patients in this study was 26.3% which was more than 8.3% reported in the Korean study [12]. The proportion of RLS sufferers among the total participants in a study done in Denmark was reported to be 5.2% which was more than 2.5% as observed in the present study [17]. The RLS sufferers constitute a medically significant group who most likely require medical treatment [18].

The present study observed that as many as 26.3% participants had the frequency of symptoms 4 or more times per week which was lesser than that reported among 50.1% patients in a study done in Pakistan [5].

The proportion of newly diagnosed RLS participants with moderate to severe degree RLS in the present study was 63.2% in comparison with 25% [12], 67.4% [5], 68.3% [15] reported in studies done at Korea, Pakistan and China, respectively.

No sociodemographic variables were associated with the severity of RLS in the present study. On the contrary, the study done in Bangalore, India, reported that RLS severity was significantly higher among males [3].

The present study observed that prolonged sleep latency and sleep disturbances were more among participants newly diagnosed with RLS. This was supported by the observation of Rangarajan and colleagues, where sleep latency was prolonged among participants with RLS [3]. RLS is, therefore, related to difficulty in initiating and maintaining sleep and disrupting sleep. In addition, a study done in Saudi Arabia reported the presence of RLS to be associated with daytime sleepiness and habitual snoring [13]. On the contrary, a Korean study reported no association between the presence of RLS and sleep profiles among the participants [12].

In the present study, subjective sleep quality was affected and sleep disturbances were more among newly diagnosed participants with severe degree RLS. In the study done in Bangalore, India severity of RLS was found to positively and significantly correlate with delay in sleep onset [3].

RLS is a major determinant of insomnia among the affected population, which drives them to seek medical care [1]. Sleep disturbances are a known determinant of stroke and other cardiovascular disorders [19]. These observations infer that improper sleep habits among the RLS patients could also affect their health and well-being, work productivity, and social activities.

The majority of previously diagnosed patients in the present study were on Ayurvedic treatment for RLS. Medications are commonly advised among primary RLS patients when symptoms are of moderate or severe degree [18].

## Conclusions

The prevalence of RLS in the present study was higher than that reported in previous Indian studies. Determinants such as family history of RLS and the presence of diabetes mellitus were independently associated with its presence, indicating the role of genetic factors and lifestyle diseases in its occurrence. Unpleasant sensations in RLS significantly affected sleep initiation and maintenance among the affected. A multi-disciplinary approach is required to control its determinants and address other sleep-related problems among the RLS affected population at this setting.

## Limitations

The cross‑sectional design might be a deterrent to establishing a causal relationship. There is also a possibility of non-reporting of symptoms by the participants. There were few participants with diabetes mellitus, hypothyroidism, and rheumatoid arthritis in study. There is a possibility that some of these patients might be also suffering from peripheral neuropathy, which is a major differential diagnosis of RLS. The non-random selection of 202 participants considering the prevailing COVID-19 conditions could also be another limitation of this study.

## Data Availability

The research data are deposited in the Figshare repository: ‘Study on restless leg syndrome and its relationship with quality of sleep among the general population of India’. https://doi.org/10.6084/m9.figshare.19550776

## Abbreviations

RLS: Restless leg syndrome
IRLSSG: International Restless Legs Syndrome Study Group Rating Scale
PSQI: Pittsburgh Sleep Quality Index
COVID-19: Coronavirus disease 2019
FBS: Fasting blood sugar
BMI: Body mass index
SES: Socioeconomic status
SPSS: Statistical Package for Social Sciences
UOR: Unadjusted odds ratio
AOR: Adjusted odds ratio
CI: Confidence intervals
SD: Standard deviation
